# Experimental Validation of Injection Molding Simulations of 3D Microparts and Microstructured Components Using Virtual Design of Experiments and Multi-Scale Modeling

**DOI:** 10.3390/mi11060614

**Published:** 2020-06-24

**Authors:** Dario Loaldi, Francesco Regi, Federico Baruffi, Matteo Calaon, Danilo Quagliotti, Yang Zhang, Guido Tosello

**Affiliations:** Department of Mechanical Engineering, Technical University of Denmark, Building 427A, Produktionstorvet, DK-2800 Kgs Lyngby, Denmark; darloa@mek.dtu.dk (D.L.); fr@rel8.dk (F.R.); federico.baruffi.91@gmail.com (F.B.); mcal@mek.dtu.dk (M.C.); danqua@mek.dtu.dk (D.Q.); yazh@mek.dtu.dk (Y.Z.)

**Keywords:** modeling, micro-injection molding, micro replication, process simulation

## Abstract

The increasing demand for micro-injection molding process technology and the corresponding micro-molded products have materialized in the need for models and simulation capabilities for the establishment of a digital twin of the manufacturing process. The opportunities enabled by the correct process simulation include the possibility of forecasting the part quality and finding optimal process conditions for a given product. The present work displays further use of micro-injection molding process simulation for the prediction of feature dimensions and its optimization and microfeature replication behavior due to geometrical boundary effects. The current work focused on the micro-injection molding of three-dimensional microparts and of single components featuring microstructures. First, two virtual a studies were performed to predict the outer diameter of a micro-ring within an accuracy of 10 µm and the flash formation on a micro-component with mass a 0.1 mg. In the second part of the study, the influence of microstructure orientation on the filling time of a microcavity design section was investigated for a component featuring micro grooves with a 15 µm nominal height. Multiscale meshing was employed to model the replication of microfeatures in a range of 17–346 µm in a Fresnel lens product, allowing the prediction of the replication behavior of a microfeature at 91% accuracy. The simulations were performed using 3D modeling and generalized Navier–Stokes equations using a single multi-scale simulation approach. The current work shows the current potential and limitations in the use of micro-injection molding process simulations for the optimization of micro 3D-part and microstructured components.

## 1. Introduction

A consolidated trend in micro-manufacturing consists of the adoption of replication technologies for large-scale productions. Due to its high throughput and overall capabilities, combined with the possibility of automating the process, micro-injection molding (µIM) is the most commonly found replication process in multiple applications and industries including medical, optical, consumer, sensors, and micro electro-mechanical systems (MEMS) [1]. To hasten the micro product design phase, optimize µIM process conditions as well as predict process quality and performance, significant attention has been dedicated to the numerical modeling and the simulation of such technology, aiming for the establishment of a µIM digital twin. The µIM process encompasses three families of products that significantly constrain the technical equipment required for processing and simulation [2], as described in Table 1. µIM process simulation has been developed from conventional IM modeling methods. Multiple commercially available software for the scope includes, for example, Autodesk Moldflow^®^ (Autodesk Inc., San Rafael, CA, USA), Moldex3D (CoreTech System Co., Hsinchu County, Taiwan), Simpoe-Mold (Dassault Systèms, Hertogenbosch, Netherlands), Sigmasoft^®^ (SIGMA Engineering GmbH, Aachen, Germany), and Rem3D^®^ (Transvalor S.A., Mougins, France). The numerical simulation is structured by solving a system of equations that enclose the conservation of mass (Equation (1)), linear momentum (Equation (2)), and energy (Equation (3)).

(1) $$\frac{d}{dt}\rho +\nabla \cdot \left(\rho v\right)=0$$

(2) $$\rho \frac{d}{dt}v=\rho g-\nabla P+\eta \nabla^{2}v$$

(3) $$\rho c_{p}\left(\frac{\partial }{\partial t}T+v\nabla T\right)=\beta T\left(\frac{\partial }{\partial t}P+\vec{v}’\times \vec{\nabla }P\right)+\nabla \cdot \left(k\nabla T\right)+\dot{\eta \gamma^{2}}$$

where *ρ* is the density; *t* is the time; *v* is the velocity vector; *g* the gravitational acceleration constant; *P* is the hydrostatic pressure; *η* is the viscosity; *c_p_* is the specific heat; *T* is the temperature; *β* is the heat expansion coefficient; *k* is the thermal conductivity; and *γ* is the shear rate. Additional boundary conditions that describe the polymer flow are added to the system, which include the polymer *pvT* constitutive relationship often described with the Tait equation, and a velocity-dependent viscosity model often described with the Cross-WLF [3]. When scaling down from the macro IM toward µIM, several additional physics should be considered (see Table 2) and a summary of their effects is discussed below.

Regarding the discretization of the solution domain, it is well known that 3D elements are necessary for decomposing micro-components when the wall thickness to flow length ratio is no longer negligible [4,5,6]. In addition, a high fidelity calibration of injection pressure and flow length can be achieved when the feeding systems (gate, runner, sprue, and injection unit as a hot runner) are included in the simulation domain [7,8]. At the microscale, the no-slip boundary condition at the wall is no longer a valid hypothesis. In fact, Cao et al. showed that for microcavities, the required pressure drop to create the polymer filling induced polymer slippage at the walls. Thus, a non-zero-velocity boundary condition should be considered for the shear stress at the walls to solve the system of equations (Equations (1)–(3)) [9,10]. Moreover, capillary effects generated by surface tension forces become relevant, especially for nanoscale cavities. In order to include the surface tension and to account for the pressure loss/gain at the polymer melt interface, Rytka et al. [11] introduced an additional force in Equation (2). In the same study [11], polymer material properties such as flow temperature and contact angles were re-engineered or re-measured and modified in the simulation software database, since small variations of these parameters have been claimed to significantly affect the simulation results at the microscale. To calibrate the µIM simulations, another parameter that differs significantly from the macro scale simulations is the local heat transfer coefficient (HTC). Depending on the velocity gradient from the walls, different shear stress and viscosity are developed, leading to a different Nusselt number and HTC. Conventional simulation approaches use an average HTC for the filling and packing phases. However, since the thermal equilibrium between the polymer melt and mold establishes much faster at the walls than at the cavity core, a local HTC is assigned to the model properties when representing microfeatures. In this way, a more accurate prediction of the skin layer formation and of the flow rate can be found [9,12,13]. 

Other physical phenomena of central importance in µIM that need to be implemented in the simulation models are the effect of imperfect venting of micro-injection molds and the consequent counter pressure induced by the residual trapped air inside the tool’s microcavity [14,15]. Furthermore, one other particularly challenging aspect for µIM process simulation is the implementation of the cavity’s surface topography into the model. Even though this term was found to be significant in altering the filling behavior of microfeatures [16], the boundary layer on all the skin parts would require a consistent extension of the number of elements and time required for the simulation. In addition, it is not cost-effective to measure the entire surface roughness of a mold cavity and correctly model it in a digital form.

When focusing on parts featuring surface micro/nano structures, the implementation of simulation protocols becomes even more challenging due to the intrinsic multiscale nature of the domain. In order to reduce computational and modeling effort, the µIM simulation is often broken down in two separated steps: first, a macro/meso scale simulation of the part cavity without surface structures, which is followed by a second micro/nano simulation of the single surface feature where additional physics are added based on the size and geometry of the features themselves. In the first step, the temperature, pressure, and velocity of the polymer melt during injection are found and fed into the second step as the boundary conditions. For nanoscale features, the size of the polymer chains can also be considered using molecular dynamics simulation approaches [17,18,19].

**Figure 1:**
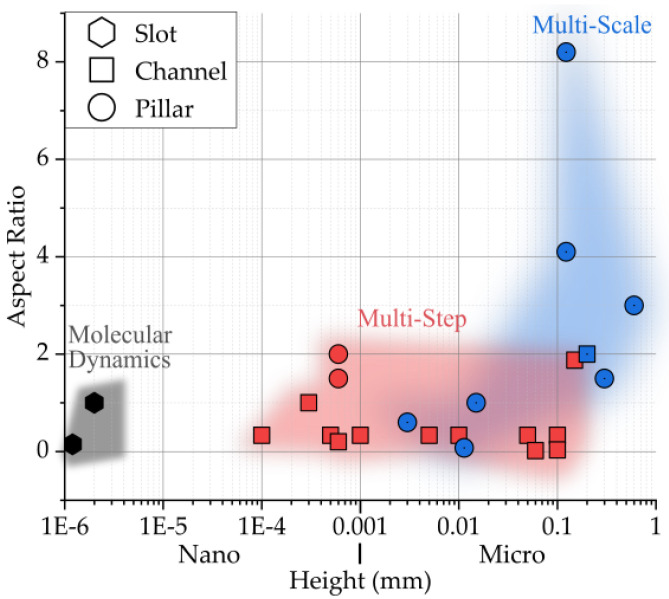
Comparison of µIM modeling cases for parts featuring micro/nano surface structures based on the structures’ aspect ratio, height, and feature geometry [9,10,11,17,18,19,20,21,22,23,24,25,26,27,28,29].

This sequential method is not scalable because it requires case-by-case multi-step validation for each boundary condition that needs to be extracted, and is fundamentally based on the assumption that the boundary conditions are providing a sufficiently good approximation as the start input for the finer model. For this reason, an integrated multi-scale approach would be preferred. With the term multi-scale, a single simulation that combines a multi-scale mesh or multi-scale model formulation is intended. In Figure 1, a case selection of modeling studies shows the employed simulation method in comparison to the feature aspect ratio and its size [9,10,11,17,18,19,20,21,22,23,24,25,26,27,28,29]. 

This work proposes four case studies in which integrated multi-scale µIM process simulations are employed as a digital process optimization tool. In the first case, the calibration of the model using the effective mold microfeature dimension was used for the selection of process parameters in single micropart production. In the second case, a full factorial design of experiments and simulations investigated how to predict flash formation in a single micropart production by adding the venting channel as part of the cavity domain. In the third and fourth cases, meshing and domain partitioning strategies were proposed. The presented approaches aimed to realize a unique integrated multi-scale method for the investigation of parts featuring low-aspect-ratio microstructures for optical applications.

## 2. Optimization of 3D Micropart Production

### 2.1. Case 1—Micro-Ring

**Figure 2:**
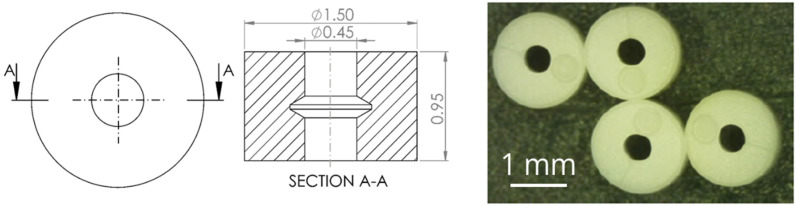
Design specification of the polymer micro-ring (**left**) and micro-molded parts (**right**) [30].

The first case under analysis focused on the implementation of the µIM process simulation for the optimization of the production of a micro-ring. The part was a ring with a nominal outer diameter of 1.5 mm and an internal diameter of 0.45 mm. The part was manufactured with a commercially available thermoplastic elastomer (TPE) (Cawiton^®^ PR 10589 F, B.V. Rubberfabriek Wittenburg, Zeewolde, the Netherlands) and its final mass was 2.2 mg. The manufacturing tolerance for the outer diameter production was 10 µm. The design of the part is reported in Figure 2. The purpose of the simulation was to predict the dimension of the outer diameter and find an optimal set of process parameters that would allow for production within the specifications.

### 2.2. Case 2—Micro-Cap

**Figure 3:**
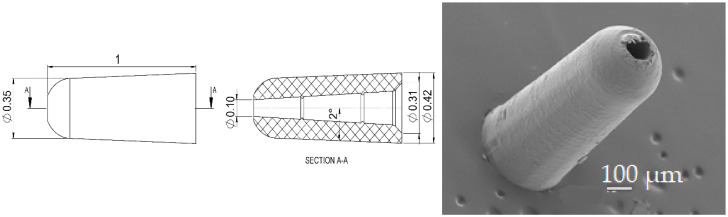
Design specifications of the polymer micro-cap in the analysis (**left** and **center**); scanning electron microscope image of the micro-injection molded part (**right**) [31].

The second case refers to another single micro-component that had a nominal part weight of 0.1 mg. The part is depicted in Figure 3, and has application in the medical industry. The part was made of a high-flowability commercially available polyoxymethylene (POM) (Hostaform C 27021, Celanese Corporation, Dallas, TX, USA) and had a hollow tapered internal geometry. The critical production aspect of the part consists of the formation of flash at the end of the part and µIM simulation was employed for the prediction and evaluation of flash formation based on a given set of process parameters.

### 2.3. Multi-Scale Modeling and Meshing of Single Micro-Components

**Figure 4:**
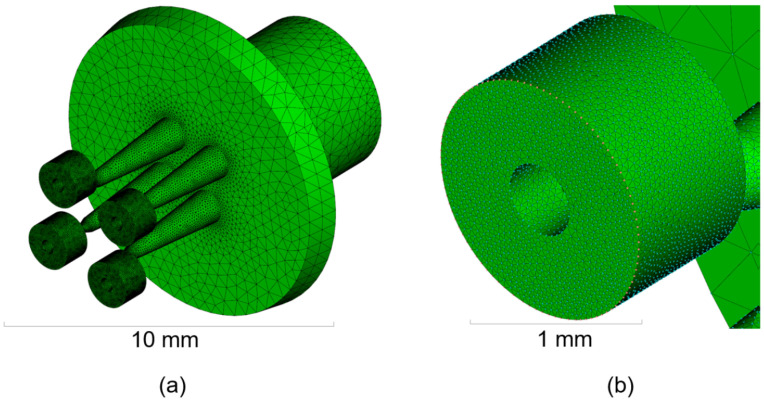
Multi-scale mesh of Case 1 including: sprue, runners, and multicavity tooling system (**left**); detailed view of the molded part and of the venting structure (**right**) [31].

**Figure 5:**
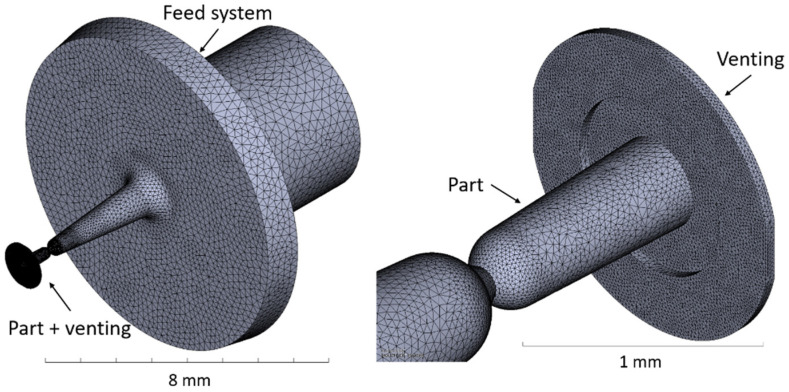
Multi-scale mesh and modified model geometry of Case 2 to include the flash formation in the simulation of the micro-cap [31].

Both simulations in Case 1 and Case 2 were implemented in Moldflow^®^ Insight 2017 (Autodesk Inc., Melbourne, Australia) software using a multi-scale approach. No additional subroutines were developed in addition to the commercially available version of the software. The mesh was generated in both cases using tetrahedral elements with varying dimensions from 50 µm to 500 µm for Case 1 (see Figure 4) and 20 µm to 300 µm for Case 2 (see Figure 5). The entire feeding system was included in the models as it takes most of the overall material to produce single components. The total number of elements handled in the simulation was 1.4 million in Case 1 and 1.0 million in Case 2. The material properties in terms of *pvT* and viscosity follow a double-domain Tait model equation and a Cross-WLF model, both available in the software library. Experimental µIM parts were produced using a MicroPower 15 machine, (Wittmann Battenfeld Vienna, Austria). A summary table of the simulation multi-scale parameters is presented in Table 3.

**Figure 6:**
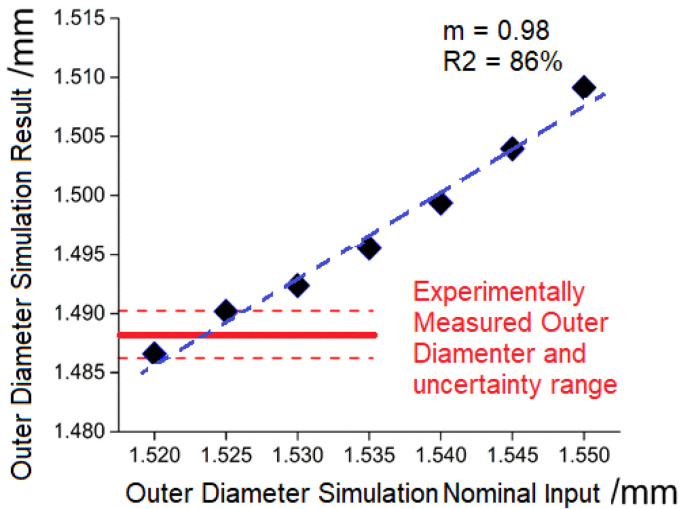
Design specification of the demonstrator with light selective reflection surface structures.

In Case 1, the measured mold diameter was used as the nominal dimension. The outer diameter of the micro-ring cavity was measured using an optical coordinate measuring machine (DeMeet 3D, Schut Geometrical Metrology, Groningen, The Netherlands). An initial validation of the model was performed by comparing the diameters of the measured part and the simulated one. An initial deviation of 21 µm was measured from the experimental and simulated results. The model validation was performed using a parametric approach. The nominal outer diameter in the simulation was systematically reduced by 5 µm and the respective simulated result was compared to the experimental result until the final deviation from the values was found to be lower than the uncertainty of the measurement of 2 µm. The total number of iterations to achieve an attuned model was six, and the individual results are reported in Figure 6. A linear correlation was found from the nominal and resulting diameter, finding a uniform deviation from the simulation input and resulted in a calculated distance of (38 ± 3) µm. This finding indicates that the systematic difference could be amended by a correction factor (0.98 in this case), achieving an accuracy below the measurement uncertainty. It was assumed that a combination of factors including the actual dimension of the other component features as well as the effective shrinkage of the material at processing conditions, influenced the magnitude of the correction factor.

In Case 2, the model design was modified in order to include flash in the molded part. This was achieved by adding an outer ring that develops radially from the component right-end of Figure 3. The modified model of the part is shown in Figure 5. The thickness of the flash domain was set in two areas, the first one spreading up to twice the cap outer diameter to 0.84 mm with a thickness of 10 µm, while the second part spread to an outer diameter of 1.26 mm with a thickness equal to 20 µm. The definition of these geometrical properties was based on the outcome of different parametric trials.

### 2.4. Process Optimization Based on Calibrated Simulation Results—Case 1

**Figure 7:**
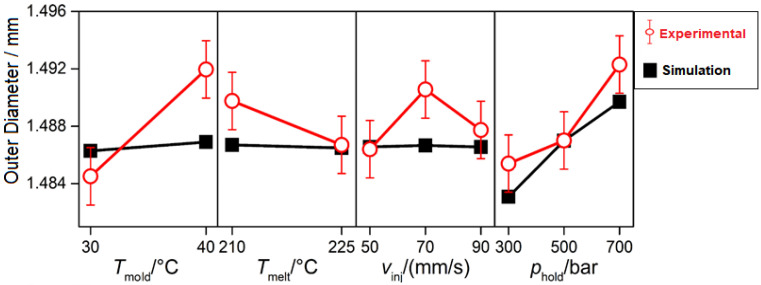
Main effects plot of the process factors on the outer diameter of the micro-ring [31].

For Case 1, the calibrated simulation model was subsequently used to run an optimization campaign based on four factors: mold temperature, melt temperature, injection velocity, and holding pressure. The temperature factors were varied on two levels, {30, 40} °C and {210, 225} °C, while velocity and pressure were varied on three levels, {50, 70, 90} mm/s and {30, 50, 70} MPa, respectively. The total number of the combinations was 2^2^ × 2^3^ = 36. The simulation results were compared with equivalent experimental analysis, which included five process replications and three repeated measurements for a total number of 540 data points. In Figure 7, the comparison of the main effects of each individual process factor on the outer diameter is reported.

**Figure 8:**
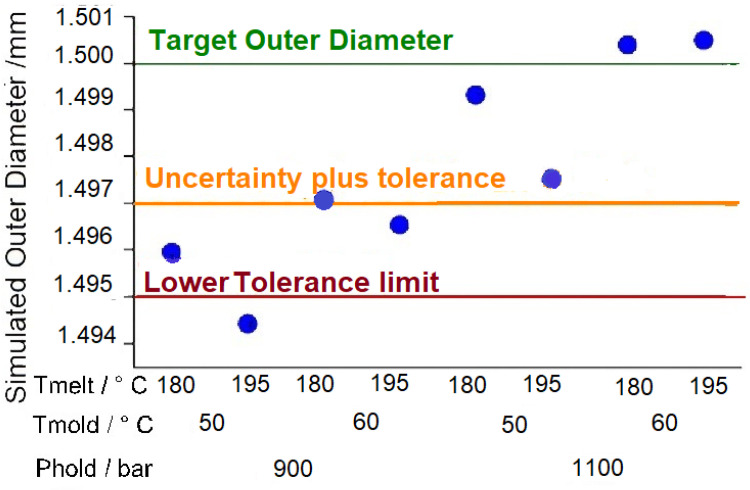
Simulated optimal process conditions for the outer diameter of the micro-ring.

The main effects showed an average deviation of (2.1 ± 2.0) µm from the simulated and experimental values with a maximum deviation of 5.5 µm observed for the cases at high mold temperature. The other cases indicated a deviation within 3 µm. These results validate the possibility of using the simulation as a calibrated tool for process optimization. As a matter of fact, an additional factorial design was simulated to find the optimal process conditions with a range of wider factor levels. There were three new factors in the analysis: mold temperature {50, 60} °C, melt temperature {180, 195} °C, and holding pressure {90, 110} MPa. The results of the investigation are shown in Figure 8, which also presents the target nominal diameter as well as the uncertainty of measurements and the tolerance limit. In this case, the µIM process simulation was able to predict that half the process conditions (those corresponding to high holding pressure, i.e., 110 MPa) investigated in the analysis would yield parts out of specification when the measurement uncertainty was considered. 

### 2.5. Optimization of the Flash Formation Using Process Simulation—Case 2

**Figure 9:**
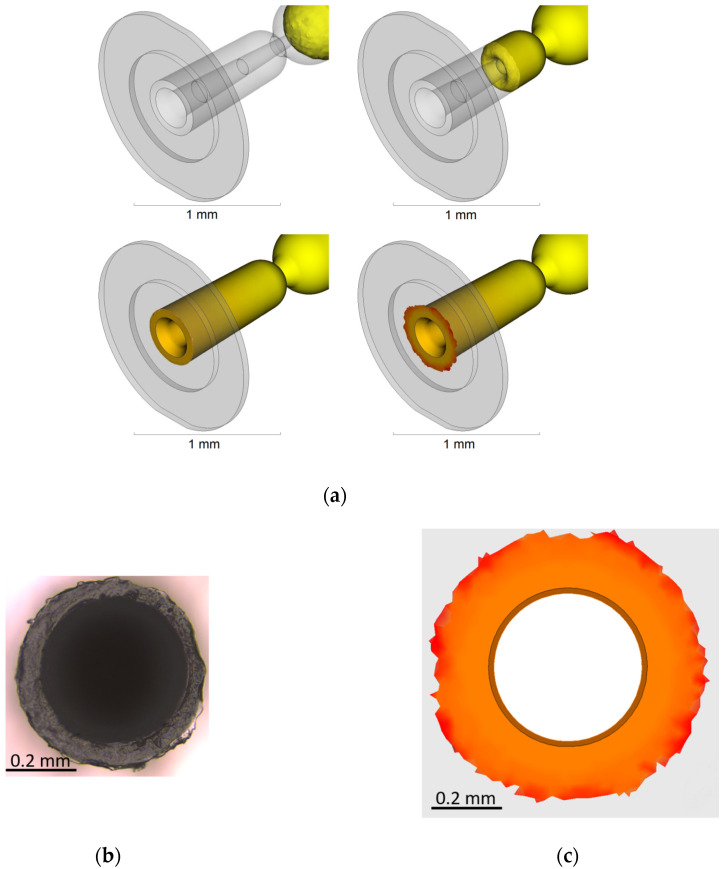
(**a**) Simulated flash formation during cavity filling; (**b**) real flash on the part; and (**c**) simulated flash (the dimensional scale bar is equal in both images) [31].

**Figure 10:**
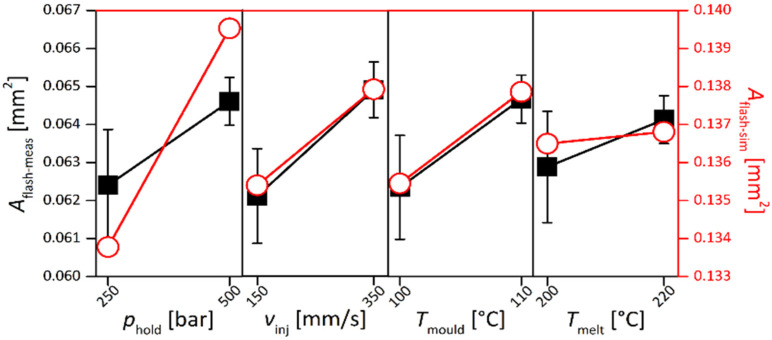
Main effects plot of the flash area for the experiments A_flash-meas_ (in black) and simulations A_flash-sim_ (in red). Note that the scales for the two sets of results are different, but the ranges shown are equal. Interval bars represent the standard errors of experimental data [31].

For Case 2, a full factorial design was performed with the following factor levels: mold temperature (100–110 °C), melt temperature (200–220 °C), injection velocity (150–350 mm/s), and holding pressure (25–50 MPa). The total number of experiments was, in this case, replicated five times and the simulation was carried out once for each process condition. The simulated flash formation at the end of the cavity filling is shown as the result of the step-by-step flow simulations in Figure 9a. The area of the experimental flash (Figure 9b) and of the simulated flash (Figure 9c) was then measured by image processing for each process combination of both the real and virtual DOE. In Figure 10, the comparison of the main effects affecting the measured flash formed area against the simulated once is reported. The scale bars of the simulated and actual experiments ranged on a scale of 7 µm. Although the nominal values of the simulated results were overestimated by a factor 2.1, in relative terms, this means that the simulation predicted a flash area that was twice as large as the actual experimental case. Nonetheless, the effect amplitude and sign were congruent for injection velocity, melt temperature, and mold temperature variations, indicating that the amplitude value could be calibrated by the previously mentioned factor to obtain an accurate prediction of the flash area for a given set of molding parameters. With this result, it is possible to use µIM process simulation to find the effect of process parameters on flash formation and at the same time find a calibration factor for the design of the microcavity vent.

## 3. Multi-Scale Filling Simulation of Low Aspect Ratio Structures in Parts Embedding Microfeatures

### 3.1. Case 3—Micro-Optical Reflector

**Figure 11:**
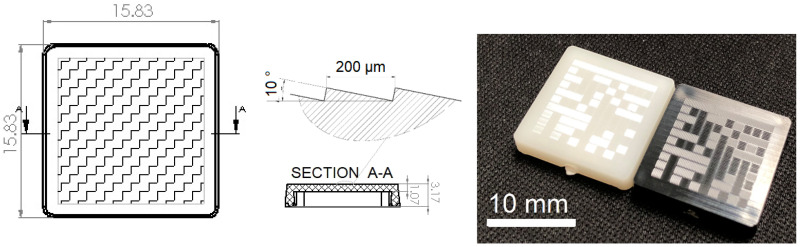
Design specification of the micro-optical reflector (**left**) with a highlight of its surface structures (**center**); injection molded part and tool cavity insert (**right**) [32].

The use of µIM process simulation was further extended to parts embedding a microstructured surface with low aspect ratio micro-grooves. The Case 3 component is an optical demonstrator with surface features that enable light selective reflection (Figure 11). The structures consist of parallel triangular grooves with a nominal height of 34 µm, a width of 200 µm, and a slope of 10°, resulting in a growing aspect ratio from 0 to 0.17. The part was molded in a commercially available acrylonitrile butadiene styrene (ABS) (Terluran^®^ GP35, INEOS Styrolution Group GmbH, Frankfurt, Germany) and the mass of the actual part was 401 mg.

### 3.2. Case 4—Fresnel Lens

**Figure 12:**
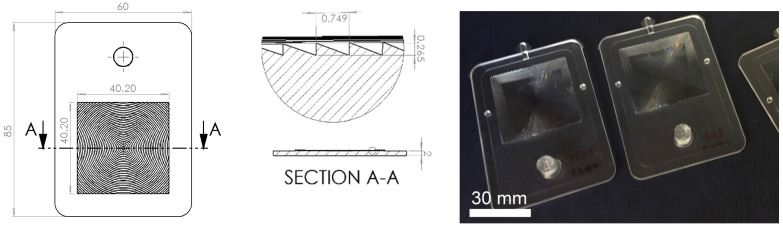
Design specification of the analyzed Fresnel lens (**left**) and detail of the low aspect ratio surface structures (**center**); injection molded parts (**right**) [33].

Case 4 is represented by a Fresnel lens whose surface is structured by low aspect ratio features. The specimen was manufactured in a commercially available cyclo-olefin polymer (COP) (Zeonex E48R^®^, Zeon Corporation, Tokyo, Japan) and had a total mass of 13.4 g. The part had global dimensions of 60 mm × 85 mm and was covered with concentric surface microgrooves. The structures were semi-pyramidal with a constant pitch of 749 µm and a varying height from 17 µm to 346 µm with a growing aspect ratio of 0.02 to 0.46 from the center to the outer of the structure array. The total array covered an area of 40 mm × 40 mm and is shown in Figure 12.

### 3.3. Multi-Scale Meshing for Microstructured Parts

Both microstructured parts exhibited a highly multi-scale nature of the design with a minimum to maximum feature dimension ratio of 34 µm (microfeature height)/15.830 mm (part edge length) (i.e., aspect ratio 1/466) for the reflector and 17 µm (minimum microfeature height)/85.000 mm (part long edge length) (i.e., aspect ratio 1/5000) for the Fresnel lens. When generating a mesh that handles this aspect ratio, it is necessary to find a compromise between the meshing parameters (minimum/maximum element size, growth rate, and aspect ratio of each element), computational time, and accuracy. Some of the previously mentioned software providers allow for hybrid mesh generation that refines the element size at the surface in order to address the surface phenomenon and features. Others allow for mesh partitioning in order to generate a local surface refinement where required. For the two proposed cases, the Autodesk Moldflow^®^ Insight 2019 (Autodesk Inc., Melbourne, Australia) software was employed for mesh generation and simulation. No additional subroutines were developed in addition to the commercially available version of the software. 3D tetrahedral elements were employed. Two different approaches were employed in order to find a compromise between the computational effort and the simulation accuracy. 

**Figure 13:**
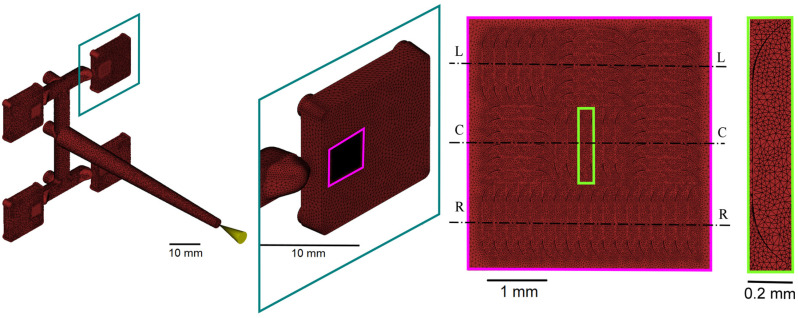
The multi-scale mesh of the optical demonstrator with selected surface features included in the geometry [35].

For Case 3, only a fraction of the microfeatures array was added to the simulation domain (i.e., in the simulated geometry). The features closer to the gate were included due to the lower replicability that was observed experimentally [32]. An advancing layer [34] algorithm was used to first generate the surface mesh elements, and subsequently in the mesh elements across the part thickness. The meshing parameters are summarized in Table 4. The part, in this case, was produced in a four cavity mold and the resulting mesh is shown in Figure 13. 

**Figure 14:**
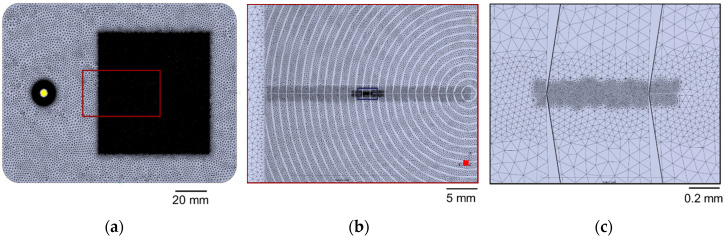
The multi-scale mesh of the Fresnel lens with partial surface mesh refinement at (**a**) part level, (**b**) structured area level, and (**c**) at single micro feature level.

For Case 4, the whole surface microfeatures were included in the modeled part geometry, but only a part of them was locally refined using surface mesh partitioning areas. The mesh was created using an advancing front [36] algorithm by first generating the surface elements. A local refinement on the surface microfeatures down to an element size of 10 µm was performed, as shown in Figure 14. Additional meshing parameters are shown in Table 4.

### 3.4. Multi-Scale Filling Simulation Validation at Mesoscale—Cases 3 and 4

**Figure 15:**
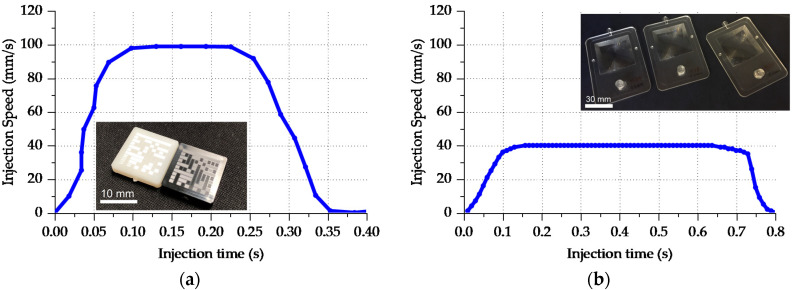
Injection speed profiles sampled from the machine controller for Cases 3 (**a**) and 4 (**b**).

Validation of the filling behavior requires an experimental comparison of injection molded parts with the simulation results. For Case 3, an Allrounder 370A injection molding machine from Arburg (Loßburg, Germany) was employed with an injection screw of 18 mm in diameter. For Case 4, the employed machine was a Ve70 from Negri Bossi (Cologno Monzese, Italy) equipped with an injection screw with a diameter of 26 mm. The injection screw absolute velocity over time and its initial position were used as input parameters for the injection molding simulation. The profiles are reported in Figure 15a for Case 3 and Figure 15b for Case 4.

**Figure 16:**
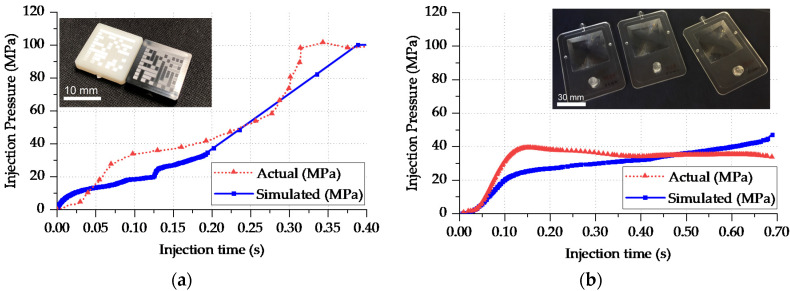
Injection pressure at the injection location comparison from the simulation and actual value for Cases 3 (**a**) and 4 (**b**).

The simulation validation for Cases 3 and 4 included the injection pressure profiles, as shown in Figure 16. The actual value was extracted directly from the injection molding machine control interface and represents the pressure in the filling phase. The validation plot shows that during filling, the actual simulated pressure required to fill the cavity was higher than the simulation. For Case 3, the simulated integral over time of the injection pressure was 20.1 MPa s against 22.0 MPa s with an underestimation of pressure over time by 1.9 MPa s, which in this case was 8% of the actual value. For Case 4, the simulated integral had a value of 40.1 MPa s against the effective 41.5 MPa s with a nominal deviation of 1.4 MPa s, which in relative terms was 3% of the actual value. The pressure underestimation was attributed to the presence of surface roughness and the absence of a full multi-scale surface microfeature domain.

### 3.5. Multi-Scale Simulation for Filling Time Prediction—Case 3

**Figure 17:**
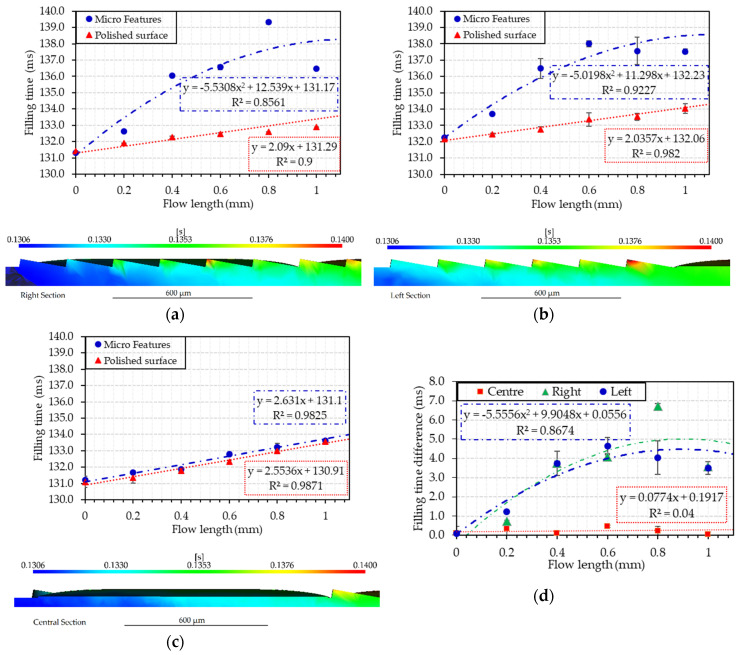
Filling time comparison from the microfeature and bottom polished surface as a function of the flow length for the right (**a**), left (**b**), and central (**c**) section of Case 3 with the summary difference of filling time for each condition (**d**).

At the microscale, the surface features were fully replicated in both of the considered design solutions. Specifically for Case 3, the restricted features under analysis were compared in terms of filling time. A total of 250 intermediate results during filling were calculated to achieve a time resolution of 0.8 ms. A filling time comparison from the top edge of the microfeature and corresponding bottom polished surface at the same X coordinate was made for the three sections shown in Figure 13 intended as left (L), central (C), and right (R). The results of filling time as function of the X coordinate (i.e., the flow length) are reported in Figure 17a–c. A comparison of residual filling time is then presented in Figure 17d. As can be seen in Figure 12a and Figure 17b, the orientation of the microstructures is orthogonal with respect to the flow propagation direction (i.e., the flow front velocity vector main direction). As a matter of fact, it can be seen that the melt flow was delayed when filling of the microfeatures occurred (Figure 17a,b) in comparison to when the flow was filling the bottom flat (i.e., unstructured, surface, see Figure 17c).

The delay followed a quadratic trend and was found for the two different and opposite sections of the flow front. In contrast, at the center, the orientation of the microfeatures is oriented longitudinally to the melt front, and as shown in Figure 17c, there is no induced delay of the filling time. The result indicates that µIM simulation can be used to predict the influence of surface microstructure arrays on melt front propagation. This result is an outcome of the continuity and conservation of linear momentum, and differently from other studies [37,38,39], neglects the wall slip. This result leads to the conclusion that surface microfeature arrays and their orientation affects the melt front propagation; leading to an even greater need for fully-integrated multi-scale models to avoid inaccurate estimation of velocity for a multi-step modeling approach. The mesh with a restricted portion of surface features allowed finding a correlation between flow length and filling time of periodic microfeatures.

### 3.6. Multi-Scale Simulations for Microfeature Replication Prediction—Case 4

**Figure 18:**
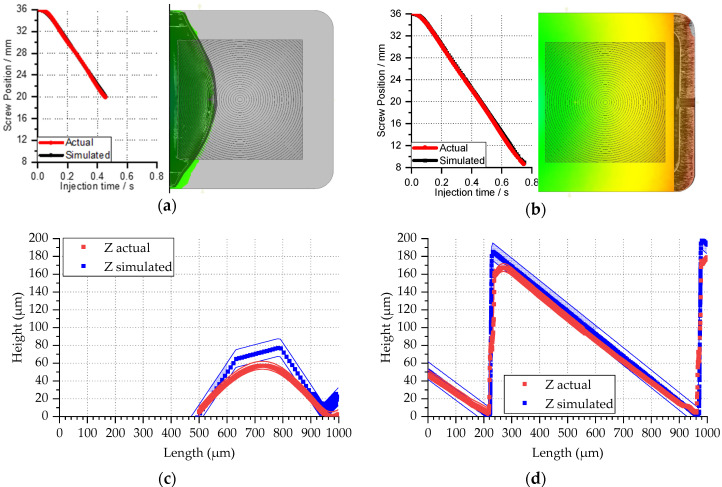
Filling volume comparison from the actual and simulated values for a short shot at meso- (**a**) and microscale (**c**), and full replication for the meso- (**b**) and microscale (**d**).

In Case 4, the multi-scale approach was employed for the evaluation of microfeature replication during the molding process. The task was solved by performing injection molding of a short shot that corresponded to a condition where a microfeature was partially replicated. The aim of this process was to show a methodology that could be used to validate the simulation with the experimental data. The evaluation of microfeatures was done experimentally by measuring shots using a laser confocal microscope (LEXT OLS4100, Olympus, Tokyo, Japan). A cross-section of the sample image was exported and considered as the reference profile. In a virtual environment, the simulated short shot was measured after the extraction of the surface elements coordinates and sectioning along the same axis. To do so, the filling results were exported in the (*.stl) format, and the surface nodal vertex belonging to the cross-section in the medial axis of the specimen was sampled and plotted to find the simulated profile of the microfeature. Two-time instances were considered for the comparison: an initial case when half the microfeature was filled corresponding to a short shot, as shown in Figure 18a,c, and for full replication, as shown in Figure 18b,d. The shot was sampled at the same injection screw displacement (16 mm) at a simulated time of 0.485 s. The melt front propagation at the mesoscale was compared between the simulation and the experiments by overlapping the top view of the simulated filling step with an optical image of the parts obtained by stitching individual confocal microscope color image acquisitions. For the full replicated part, the same validation at the mesoscale was conducted. Parts were analyzed comparing the flow front final length and injection stroke (28 mm) at an injection time of 0.752 s. At the microscale, the feature on the short shots had different height values in terms of peak-to-valley measurements. The simulated feature height was 79 ± 10 µm and the measurements on the molded feature led to 56.9 ± 4.7 µm. The simulated value for the full replication step was equal to the nominal dimension 185.0 ± 10 µm (i.e., 100% replication), while the actual value was in fact 169.0 ± 4.7 µm (i.e., 91% replication). The tip radius of the measured feature (55 µm) was a combination of both the actual mold finite edge, which for the metrological limitation and implementation on the full scale of the simulation model were not included, and the replication factor of the molding process. 

## 4. Conclusions

The current work has shown the current state-of-the-art in the use of µIM simulation capabilities. In the study, the progress of multi-scale methods against multi-step approaches was further explained by use cases for single three-dimensional microparts and parts embedding surface microstructures. From the experimental analysis and the corresponding simulation validation conducted on these cases, the following conclusions can be obtained:

- Process simulation requires geometrical calibration of the domain by measuring the effective feature size on the mold insert and feeding this value to the simulation boundary condition.
- µIM process simulation can be used for the optimization of single-part production with a 1 mm feature dimension at a 10 µm accuracy level.
- µIM process simulation can be used in the prediction of the factors most affecting flash formation in single micropart production. The punctual estimation of the flash area requires further calibration of the model geometry and a venting flow volume has to be included in the part design.
- Virtual design of experiments using simulations is an effective digital optimization tool that has the capability to indicate the effect of µIM process parameters on micro-molded part characteristics.
- For parts featuring microfeatures, two methods were proposed for the modeling of complex microstructure arrays: (1) feature restriction and (2) feature refinement.
- The feature restriction approach allowed us to model the filling time delay from the flat polished and microstructured side of the cavity, allowing us to predict the influence of feature orientation on the melt front propagation.
- Feature refinement allowed us to punctually investigate the replication development of a single microfeature. Through combined meso- and micro-dimensional scale comparison, a multi-scale validation approach was proposed.

## Figures and Tables

**Table 1:** Conventional product classification for micro-injection molding (µIM).

µIM Product	Average Part Size	Part Mass	Dimensional Tolerance Range	Equipment
Single Microparts	<10 mm	0.0001–0.1 g	10 µm	µIM metering and dosing system
Parts featuring micro- or nanostructures	>10 mm	> 0.1 g	0.01–1 µm (on features)	Conventional IM injection system
Micro precision IM Parts	>10 mm	> 0.1 g	10–100 µm	µIM and IM systems

**Table 2:** Modeling governing equations and aspects for injection molding (IM) simulation depending on scale size.

Macro/Meso	Micro (µ)	Nano (n)
Conservation of Mass	Wall-Slip Effect	Molecular Dynamics
Conservation of Momentum	Surface Tension	
Conservation of Energy	Local HTC	
Polymer constitutive equation (*pvT*)	Unvented air	
Viscosity model	Surface Roughness	

**Table 3:** Multi-scale meshing parameters for the single micro-plastic components.

Mesh Parameters	Case 1 Micro-Ring	Case 2 Micro-Cap
Element type	3D Tetrahedral	3D Tetrahedral
Meshing algorithm	Advancing Front	Advancing Front
Modeling of sprue	Yes	Yes
Number of cavities	4	1
Minimum element size	50 µm	20 µm
Maximum element size	500 µm	300 µm
Growth rate	1.2	1.2
Total elements	1.4 × 10^6^	1.0 × 10^6^

**Table 4:** Multi-scale meshing parameters for the micro-structured plastic parts.

Mesh Parameters	Case 3 Micro-Optical Reflector	Case 4 Fresnel Lens
Element type	3D Tetrahedral	3D Tetrahedral
Meshing algorithm	Advancing Layer	Advancing Front
Meshing approach	Feature restriction	Partition refinement
Minimum element size	10 µm	10 µm
Maximum element size	1.000 mm	1.000 mm
Growth rate	1.2	1.5
Total elements	3 047 407	3 248 186
